# A Review on Enhancing the Life of Teeth by Toothpaste Containing Bioactive Glass Particles

**DOI:** 10.1007/s40496-024-00366-3

**Published:** 2024-02-16

**Authors:** P. Syam Prasad, Mahammod Babar Pasha, R. Narasimha Rao, P. Venkateswara Rao, Narayanan Madaboosi, Mutlu Özcan

**Affiliations:** 1 Department of Physics, National Institute of Technology Warangal, Warangal, 506004 Telangana India; 2 Center of Dental Medicine, Clinic of Chewing Function Disturbances and Dental Biomaterials, University of Zurich, Zurich, Switzerland; 3 Department of Mechanical Engineering, National Institute of Technology Warangal, Warangal, 506004 Telangana India; 4 Department of Physics, The University of the West Indies, Mona Campus, Kingston, Jamaica; 5 Department of Biotechnology, Indian Institute of Technology Madras, Chennai, 600 036 India

**Keywords:** Bioactive glass, Dental caries, Dental materials, Oral health, Remineralization, Toothpaste

## Abstract

**Purpose of Review:**

Dental caries or tooth decay is one of the communal problems in the world which can affect not only the oral health but also the general health conditions. The main objective of this systematic review is to explore the efficacy of bioactive glass-based toothpastes against cariogenic bacteria.

**Recent Findings:**

Bioactive glass particulates containing toothpaste show better remineralization potential on demineralized enamel and dentin when compared with toothpaste containing various bioactive constituents such as fluoride and potassium chloride. These constituents in conventional toothpaste can rapidly streak off due to acidic impact in the oral environment as the bioactive glass provides minerals for demineralized enamel and dentin by forming a strong hydroxyapatite (HAp) layer on its surface. Further, the therapeutic ions present in the bioglass can resist plaque formation by raising the pH of the surrounding environment or saliva and create amicable media for healthier teeth.

**Summary:**

Toothpaste containing bioactive glass particles undoubtedly displayed the remineralizing potentiality of the dental hard tissues. Dynamics of the mineralization through different bioactive glass materials needs further investigations. In order to prevent dental cavities and improve oral health, it is important to identify and study different effective bioglass particles in toothpaste.

## Introduction

Maintaining optimal dental health goes beyond being a mere physical necessity; it stands as a cornerstone of overall well-being, shaping individuals’ ability to chew and speak, and even influencing self-esteem and social interactions. Moreover, dental challenges are ubiquitous across age groups, impacting more than just physical comfort they delve into emotions and psychological well-being. Dental ailments such as dental caries, enamel erosion, and sensitivity effortlessly affect individuals of all ages, posing significant challenges to achieving and maintaining oral health [1]. These dental problems often arise from dietary habits, brushing practices, beverage consumption, and various lifestyle factors that impact oral health [2].

Among these challenges, dental caries stands out as a global oral disease of paramount concern. Dental caries, often referred to as tooth decay, is one of the most common and significant dental problems [3]. This condition involves the erosion of tooth structure, triggered by factors such as dietary habits and the consumption of sugary and acidic beverages. The oral cavity, which houses a diverse community of microorganisms, also harbors bacteria including Streptococcus mutans and Lactobacilli that thrive on the sugars found in the dietary [4]. As these bacteria break down these sugars, they release acids as byproducts. Primarily lactic and acetic acids, these acids create an acidic environment in the mouth, resulting in a decrease in the pH level. This drop in pH (≤ 5.5) initiates a process called demineralization, wherein the mineral content of the tooth's outer layer, the enamel, is gradually dissolves. As the enamel weakens, the tooth becomes vulnerable to agents that cause decay [5]. Furthermore, the acidic environment provides a conducive setting for the growth and proliferation of harmful bacteria, notably Streptococcus mutans, which play a pivotal role in the development of dental caries [6]. Consistently consuming foods and drinks high in sugars and acidity creates an environment conducive to enamel erosion and the multiplication of bacteria that contribute to decay. This cycle of demineralization and remineralization, where minerals are deposited back onto the teeth, can be disrupted when demineralization outweighs remineralization due to dietary choices [7]. Over time, this erosion weakens the tooth’s structure, potentially leading to cavities, discomfort, and even tooth loss if not addressed.

Similar to the challenges posed by dental caries, another facet of dental health is dentin hypersensitivity (DH). Impacting approximately 10–30% of adults, DH arises due to exposed dentin surfaces, resulting in discomfort [8]. This condition, also referred to as tooth sensitivity, occurs when the protective enamel layer of the teeth becomes compromised, revealing the underlying dentin layer. Dentin is composed of tiny tubules that extend to nerve endings within the tooth. When these tubules are exposed, external stimuli by hot, cold, sweet, or acidic items can activate nerve responses, leading to a sudden, fleeting sensation of sharp pain [9]. That is, this discomfort is mainly a result of shifts in the oral environment, encompassing factors like temperature, pressure variations, solute concentrations, and local charges, which can alter fluid flow within the dentinal tubules, thus inducing painful symptoms. This discomfort can range from mild to intense and significantly affect a person’s quality of life [10]. It can curtail their choices of foods and beverages, disrupt their oral hygiene routines, and cause unease during everyday tasks such as brushing and flossing.

**Fig. 1:**
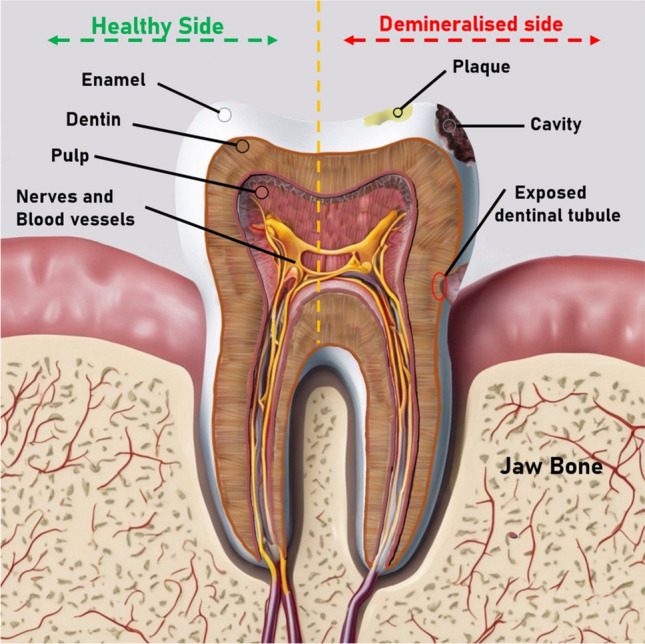
Common dental issues in the tooth

Managing dentin hypersensitivity involves a combination of preventive measures and treatment options. The hydrodynamic theory, elucidating the mechanism behind DH, suggests that stimuli applied to exposed dentin can heighten fluid flow within the dentinal tubules, triggering a mechanoreceptor response [11••]. According to this theory, two approaches for treating DH exist: (i) occlusion of open tubules: coating the dentin surface to block the tubules [12] and (ii) desensitizing therapies: targeting the pulpal nerves responsible for pain [13]. Various products such as fluorides, oxalates, varnishes, adhesive resins, sealants, lasers, Portland cement, and bioglass have been investigated to obstruct open dentinal tubules on root surfaces. Current research predominantly concentrates on reducing dentin permeability through tubule occlusion, a significant strategy for addressing tooth sensitivity [14]. Figure 1 showing the after mentioned dental issues on tooth.

Dental hygiene practices have evolved significantly from rudimentary methods to the sophisticated formulations available today. Among these, toothpaste stands as a cornerstone of daily oral care, playing a pivotal role in this ongoing pursuit [15]. Over time, toothpaste formulations have progressed from basic abrasive agents to intricate compositions tailored to address a wide array of dental needs. However, tackling the multifaceted challenges of oral health requires a more comprehensive approach beyond toothpaste alone. In this context, toothpastes containing bioactive glass have garnered substantial attention due to their promising potential in achieving tubule occlusion, consequently alleviating sensitivity [16••, 17].

Bioactive glass particles possess a remarkable set of properties that enable them to interact effectively with living tissues, rendering them transformative in the realm of oral care [18]. Their attributes of biocompatibility, osteoconductivity, and bioactivity position them as ideal candidates for various dental applications [19]. Bioactive glass has unique capability to release essential ions such as calcium, phosphate, and fluoride makes it exceptionally suited for facilitating remineralization [20]. This distinctive property propels its seamless integration into toothpaste formulations, offering the prospect of enhanced remineralization, fortified enamel, and diminished tooth sensitivity [21•].

The primary objective of this review is to comprehensively explore the potential benefits and challenges associated with toothpaste enriched with bioactive glass particles. By conducting an in-depth analysis of existing literature, clinical studies, and scientific insights, we aim to gain a thorough understanding of how bioactive glass can effectively enhance dental health. Subsequent sections will delve into the intrinsic properties of bioactive glass, its intricate interactions with tooth structure, the profound significance of dental health, and the potential advantages inherent in utilizing toothpaste containing bioactive glass. Moreover, we will examine crucial aspects such as formulation considerations, evidence gleaned from clinical investigations assessing toothpaste efficacy, safety considerations, and the potential future trajectories in this evolving field.

As dental care continues to evolve, exploring novel approaches to amplify the benefits of oral hygiene practices becomes crucial. The integration of bioactive glass particles into toothpaste formulations has the potential to revolutionize the field, offering a holistic approach to dental health maintenance. By addressing key dental concerns through enhanced remineralization and enamel strengthening, bioactive glass-containing toothpaste could transform preventive dental care. This review aims to shed light on this innovative approach and foster a deeper understanding of its implications for enhancing dental health.

## Bioactive Glass Particles in Dentistry

Bioactive glass, an intriguing material characterized by its unique properties, has garnered substantial attention for its potential applications in dentistry. Comprising a combination of silicon (Si), calcium (Ca), sodium (Na), and phosphorus (P) oxides, among others, bioactive glass is carefully engineered to mimic the mineral composition of natural bone tissue [22]. This compositional harmony imbues it with a biocompatible nature, allowing for seamless interactions with biological systems, making it an ideal candidate for integration into dental products such as toothpaste [23••].

Bioactive glass comes in various compositions, with prominent variant being 45S5 Bioglass, comprised of silicon dioxide (SiO_2_): 45%, sodium oxide (Na_2_O): 24.5%, calcium oxide (CaO): 24.5%, and phosphorus pentoxide (P_2_O_5_): 6% [24]. This variant, in particular, holds significant relevance within dentistry. Its application spans a range of dental solutions, from restorations to bone graft materials. The variations in compositional makeup intricately govern the material's bioactivity, degradation rate, and mechanical properties, effectively aligning them with their intended functions [25].

Upon interaction with oral fluids, bioactive glass particles engage in a captivating and intricate phenomenon termed bioactivity [26]. This process culminates in the creation of a hydroxyapatite (HAp) layer, a calcium phosphate compound (Ca_3_(PO_4_)_2_), within the tooth structure. This intricate process involves ion exchange and chemical reactions: upon contact with oral fluids, bioactive glass particles release sodium ions (Na^+^) into the fluid, while hydrogen ions (H^+^) from the fluid migrate into the glass structure. This exchange of ions prompts the creation of a silica-rich layer, commonly termed a silica gel, on the glass surface. This gel-like layer acts as a catalyst, initiating the subsequent formation of hydroxyapatite [26].

**Fig. 2:**
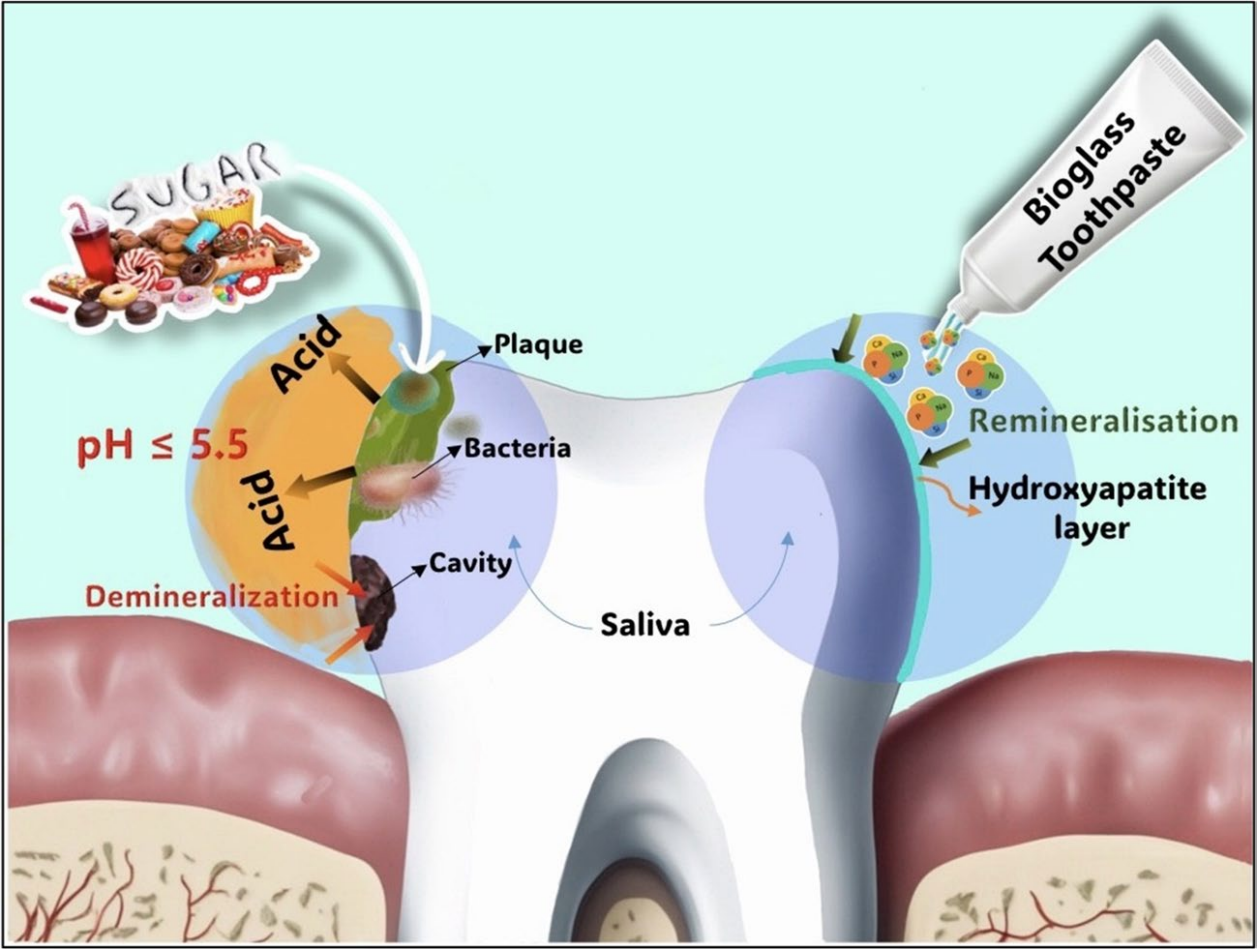
Remineralisation process of tooth with the aid of bioglass containing toothpaste

The process of hydroxyapatite formation begins with the deposition of hydroxycarbonate apatite (HCA), which serves as a scaffold for the crystallization of hydroxyapatite. This step closely emulates the mineral composition of natural enamel, promoting remineralization. Rapid release of sodium ions is accompanied by the swift substitution of hydrogen cations (H^+^ or H_3_O^+^). This ion release facilitates the subsequent liberation of calcium ions (Ca^2+^) and phosphate ions (PO_4_^3-^) from the glass particles. These initial reactions occur within seconds of exposure to an aqueous environment and persist as long as the particles remain immersed. Consequently, due to the released sodium ions, a localized and transient increase in pH occurs, triggering the precipitation of calcium and phosphate ions, ultimately resulting in the formation of a calcium phosphate layer (Ca_3_(PO_4_)_2_). Over time, as particle reactions persist and the deposition of calcium and phosphorus complexes continues, the formed layer undergoes crystallization and evolves into hydroxyapatite Ca_5_(PO_4_)_3_(OH) [19]. This hydroxyapatite shares chemical and structural similarities with the biological apatite naturally found in teeth. Figure 2 illustrating the demineralization process and mineralization process with the aid of bioglass particle containing toothpaste.

The integration of bioactive glass particles into toothpaste formulations offers compelling benefits for addressing dental caries and dentin hypersensitivity. In the context of dental caries, bioactive glass-based toothpaste aids remineralization by releasing essential ions such as calcium and phosphate [27]. This contributes to the restoration of weakened enamel and enhances tooth resistance against acid attacks. Moreover, the inherent antibacterial properties of bioactive glass particles contribute to the creation of a protective hydroxyapatite layer, effectively shielding teeth from harmful bacterial activities. On the other hand, regarding dentin hypersensitivity, the bioactive glass’s ability to deposit hydroxyapatite-like layers on exposed dentinal tubules aids in occluding tubules and reducing nerve exposure [28]. This provides relief from the discomfort associated with DH by preventing external stimuli from triggering nerve responses within the dentin.

Over the decades, the toothpaste is serving as a fundamental tool in maintaining oral hygiene, playing a crucial role in safeguarding dental health. Its daily use is pivotal for upholding oral well-being by combating various dental issues that can compromise overall health. Among the prevalent dental concerns, tooth decay, enamel erosion, and sensitivity pose significant challenges. Toothpaste, enriched with an array of essential components and ingredients, plays a vital role in preventing and managing these dental problems. Commonly containing fluoride, toothpaste strengthens enamel and guards against acid attacks, reducing the risk of cavities [29•]. Abrasives, such as silica, contribute to plaque removal and stain prevention, maintaining dental aesthetics [30]. Moreover, toothpaste formulations often include desensitizing agents, like potassium nitrate, to alleviate sensitivity, providing relief to individuals who experience discomfort from hot, cold, or sweet stimuli [23••]. However, tackling the multifaceted challenges of oral health requires an approach beyond conventional toothpaste.

In the realm of innovative dental care, the integration of bioactive glass particles into toothpaste formulations emerges as a promising solution. Bioactive glass introduces a new dimension to toothpaste functionality. The amalgamation of bioactive glass particles into toothpaste not only enhances its preventive capabilities against tooth decay, enamel erosion, and sensitivity, but also presents a holistic approach to oral health maintenance. This innovative avenue holds the potential to revolutionize traditional oral hygiene practices, contributing to a brighter and healthier dental future.

## Formulation and Delivery of Bioactive Glass Toothpaste

Several bioactive glass-based toothpaste variants have emerged, each tailored to specific dental needs. Notable examples include NovaMin [31] and BioMin [32••] toothpaste. NovaMin toothpaste primarily consists of calcium sodium phosphosilicate, an active ingredient that releases essential ions for remineralization [33]. Similarly, BioMin toothpaste incorporates bioactive glass and fluoride, delivering a controlled release of fluoride ions alongside essential ions [34]. These toothpastes leverage the unique properties of bioactive glass to provide targeted dental benefits, from remineralization to desensitization. Bioactive glass based toothpastes have been shown to be clinically effective in treating dental caries and DH and are likely to be more effective than other treatments that use primarily calcium carbonate to occlude the dentin tubules, because of the lower acid solubility of HCA compared to calcium carbonate [35]. The release of Ca^2+^ and PO_4_^3-^ ions is also useful for remineralizing incipient caries lesions and other potential benefits include an antigingivitis role [36••].

Creating toothpaste formulations that effectively harness the benefits of bioactive glass particles is a task that requires careful consideration and scientific finesse. The fusion of these microscopic powerhouses with conventional toothpaste ingredients is not without challenges. The formulation process involves a delicate balance of factors such as particle size, concentration, and compatibility, all while ensuring optimal performance and safety.

One of the fundamental challenges lies in determining the appropriate particle size and concentration of bioactive glass within the toothpaste matrix. The size of the particles plays a pivotal role in their dispersibility and interaction with other components. Too large, and they might compromise the toothpaste's texture; too small, and they might clump together or fail to provide the desired benefits. Achieving the right balance is crucial for an effective formulation. Likewise, the concentration of bioactive glass particles must be carefully calibrated. Too little, and the intended positive effects might be diluted; too much, and the toothpaste's physical properties and overall performance might be compromised. This intricate balancing act requires thorough testing to ascertain the optimal concentration that yields the desired oral health benefits without compromising the product’s integrity.

The amalgamation of bioactive glass particles with the myriad of other toothpaste ingredients is a challenge that demands a nuanced approach. The compatibility of bioactive glass with components such as abrasives, binders, flavoring agents, and preservatives must be rigorously assessed. Ensuring that the particles do not react adversely with these elements while retaining their bioactive properties is of utmost importance.

Multiple methods are explored to effectively incorporate bioactive glass particles into toothpaste formulations. One such approach involves directly mixing the particles with other toothpaste ingredients during formulation. Alternatively, nanotechnology offers a promising avenue for enhancing the delivery and dispersion of bioactive glass particles. Nanoparticles of bioactive glass can be encapsulated or coated, allowing controlled release and targeted delivery. This approach not only optimizes the utilization of bioactive glass but also enhances its interaction with enamel surfaces.

Nanotechnology presents a groundbreaking opportunity for revolutionizing bioactive glass toothpaste delivery. The manipulation of particle size at the nanoscale grants unique advantages, including improved penetration, increased surface area, and enhanced bioactivity. Nanoparticles, when encapsulated or coated, can provide sustained release of bioactive ions, extending their benefits over time. This not only optimizes remineralization but also opens doors to tailoring the toothpaste’s action to specific dental concerns.

## Clinical Studies and Efficacy

The realm of oral care has witnessed a paradigm shift with the advent of bioactive glass-containing toothpaste. A treasure trove of clinical studies has delved into its efficacy, shedding light on its transformative potential. By analyzing a selection of these studies, we unveil a tapestry of positive outcomes that underscore the tangible benefits of this innovative oral care solution.

Clinical investigations have consistently revealed the remarkable impact of bioactive glass toothpaste on reducing tooth decay rates. Findings from diverse studies have showcased a significant decrease in cavity formation when compared to conventional toothpaste. One notable study spanning a year-long observation period observed a substantial 30% decrease in new cavities among participants who diligently used bioactive glass toothpaste. This reduction reflects the potent action of bioactive glass particles in bolstering enamel resistance against the corrosive forces of decay.

The efficacy of bioactive glass toothpaste in promoting enamel remineralization emerges as a triumph in clinical research. Through meticulous examination using advanced imaging techniques, researchers have documented a discernible increase in mineral density within enamel structures. Quantitative data underscores the notable rise in calcium and phosphate incorporation, indicative of enamel’s rejuvenation. Studies have also showcased an accelerated remineralization process, offering a promising avenue for combating early-stage enamel erosion.

Beyond quantitative metrics, the true measure of bioactive glass toothpaste's efficacy lies in the tangible improvements witnessed by patients. Numerous studies have showcased positive patient feedback, ranging from decreased sensitivity-related discomfort to improved oral health indicators. Participants often laud the innovative approach for delivering perceptible results, reinforcing their commitment to using bioactive glass toothpaste as a cornerstone of their oral care routine.

## Safety and Biocompatibility

The safety of bioactive glass toothpaste hinges on meticulous research that probes potential risks associated with oral exposure. Rigorous investigations have been conducted to ascertain the interactions of bioactive glass particles with oral tissues, ensuring that their presence poses no harm. Key concerns include the potential for abrasiveness, irritability, or adverse reactions. A multitude of biocompatibility studies have been undertaken to unravel the intricate dynamics between bioactive glass particles and oral tissues. These studies involve subjecting these particles to conditions that mirror real-world exposure, closely mimicking the dynamics of oral environments. Through comprehensive analyses of cell viability, inflammatory responses, and histological assessments, researchers have garnered invaluable insights into the effects of bioactive glass particles on oral health.

The collective evidence gleaned from these biocompatibility studies points toward a generally favorable safety profile of bioactive glass-containing toothpaste. The particles exhibit a remarkable affinity for integration into oral structures, harmonizing with the oral environment rather than triggering adverse reactions. Studies consistently highlight the absence of significant cytotoxicity or adverse cellular responses. Moreover, bioactive glass particles have been found to facilitate natural healing processes, fostering cellular growth and regeneration.

Current research serves as a steadfast compass, guiding us toward the conviction that bioactive glass toothpaste is not just efficacious but also safe for oral use. The meticulous attention bestowed upon biocompatibility studies has illuminated a path paved with reassurance, where bioactive glass particles contribute to oral wellness without posing substantial risks. As the body of knowledge continues to expand, the future of oral care appears increasingly promising, where innovation and safety are intertwined in the pursuit of holistic oral well-being.

## Future Directions and Challenges

The world of bioactive glass-infused toothpaste is a promising one, yet it also presents certain challenges. The potential of bioactive glass toothpaste lies in its adaptability to various dental conditions. Crafting formulations targeted at specific challenges holds great promise. By refining aspects like particle size and composition, we can personalize oral care to combat enamel erosion, cavities, or even enhance remineralization for individuals with weakened enamel. This tailored approach ensures more effective results.

Combining bioactive glass particles with state-of-the-art delivery methods could revolutionize effectiveness. Nanotechnology’s ability to manipulate particle size and encapsulation offers untapped potential. By extending the release of bioactive ions, we can prolong oral care benefits, fortifying the remineralization process and reinforcing enamel against decay and erosion.

The journey towards bioactive glass toothpaste is not without challenges. Ensuring the lasting stability of these particles within toothpaste formulations is a key concern. Maintaining their potency and bioactivity over time, while preserving the physical attributes of toothpaste, requires careful investigation. As bioactive glass toothpaste becomes more prevalent, the challenge of production scalability arises. Ensuring consistent quality and quantity of bioactive glass particles across production batches demands robust manufacturing processes. Moreover, adhering to rigorous safety and efficacy standards in accordance with regulations is essential to establish the product’s credibility. The future of bioactive glass toothpaste holds promise for innovation and precision. It envisions formulations tailored to address specific dental needs, empowered by advanced delivery techniques. Despite challenges, this journey is filled with possibilities, where science and creativity collaborate, reshaping the world of oral care for a brighter and healthier future.

## Conclusions and Future Perspectives

Bioactive glass-containing toothpaste stands as a catalyst for transforming oral health practices, marked by its unique composition of silicon, calcium, sodium, and phosphorus oxides. The potential of bioactive glass toothpaste shines through its ability to extend tooth longevity, fortified by improved remineralization and enamel strength, safeguarding against decay and erosion. Beyond the surface, bioactive glass toothpaste offers a holistic approach by addressing tooth decay, enamel erosion, and sensitivity, propelled by essential ion release and hydroxyapatite formation. This toothpaste represents more than a routine; it has an emblem of innovation and science in action, steering us toward a future of healthier smiles and improved overall oral well-being. As we close this exploration, the horizon gleams with possibilities. Bioactive glass toothpaste holds the promise to redefine oral care, underscoring a vision of enduring dental health.
